# When the brain changes its mind: Oscillatory dynamics of conflict processing and response switching in a flanker task during alcohol challenge

**DOI:** 10.1371/journal.pone.0191200

**Published:** 2018-01-12

**Authors:** Lauren E. Beaton, Sheeva Azma, Ksenija Marinkovic

**Affiliations:** 1 Department of Psychology, San Diego State University, San Diego, California, United States of America; 2 Athinoula A. Martinos Center for Biomedical Imaging, Massachusetts General Hospital, Charlestown, Massachusetts, United States of America; 3 Department of Radiology, University of California, San Diego, La Jolla, California, United States of America; University of Verona, ITALY

## Abstract

Despite the subjective experience of being in full and deliberate control of our actions, our daily routines rely on a continuous and interactive engagement of sensory evaluation and response preparation streams. They unfold automatically and unconsciously and are seamlessly integrated with cognitive control which is mobilized by stimuli that evoke ambiguity or response conflict. Methods with high spatio-temporal sensitivity are needed to provide insight into the interplay between automatic and controlled processing. This study used anatomically-constrained MEG to examine the underlying neural dynamics in a flanker task that manipulated S-R incongruity at the stimulus (SI) and response levels (RI). Though irrelevant, flankers evoked automatic preparation of motor plans which had to be suppressed and reversed following the target presentation on RI trials. Event-related source power estimates in beta (15–25 Hz) frequency band in the sensorimotor cortex tracked motor preparation and response in real time and revealed switching from the incorrectly-primed to the correctly-responding hemisphere. In contrast, theta oscillations (4–7 Hz) were sensitive to the levels of incongruity as the medial and ventrolateral frontal cortices were especially activated by response conflict. These two areas are key to cognitive control and their integrated contributions to response inhibition and switching were revealed by phase-locked co-oscillations. These processes were pharmacologically manipulated with a moderate alcohol beverage or a placebo administered to healthy social drinkers. Alcohol selectively decreased accuracy to response conflict. It strongly attenuated theta oscillations during decision making and partly re-sculpted relative contributions of the frontal network without affecting the motor switching process subserved by beta band. Our results indicate that motor preparation is initiated automatically even when counterproductive but that it is monitored and regulated by the prefrontal cognitive control processes under conflict. They further confirm that the regulative top-down functions are particularly vulnerable to alcohol intoxication.

## Introduction

Voluntary behavior has been traditionally viewed as emerging from a serial engagement of modular processing stages starting with stimulus evaluation (“input”) carried out by the perceptual system. The regulative control network then determines the most adaptive course of action and it activates the motor system to execute the selected motor action (“output”) [1, 2]. However, extensive evidence calls into question a clear distinction in timing between sensory and motor systems [3] suggesting that these processing streams proceed largely automatically and in a parallel and interdependent manner. Some accounts propose that motor preparation of the voluntary muscles commences early during perceptual processing, once the sensory analysis delineates a possible range of motor actions most likely to be relevant in that context [4]. This preparatory activity is automatic and not accessible to conscious experience [5–7] yet it seems to underlie a significant portion of our behavior [8]. This view is inconsistent with our subjective experience of being in control of our actions, which we perceive as being fully voluntary and deliberate. Indeed, automatic processing is contrasted with cognitive control [9–11], which refers to the capacity to monitor environmental demands and flexibly select goal-directed actions. It is an executive function, it is under conscious control, and it is primarily engaged when we encounter stimuli that are ambiguous or that evoke incompatible response tendencies [12]. It is subserved by an interactive network that primarily includes medial and lateral prefrontal and lateral parietal cortices that seem to play overlapping and only partially distinct roles [8, 13–17]. Cognitive control is typically studied with tasks contrasting different levels of response incompatibility. It comprises detection of incongruity, employment of top-down control to decide between multiple options, inhibition of a dominant and automatic but task-irrelevant response, and execution of the most adaptive and goal relevant action [18–20]. Even though automatic processing underlies a large portion of our daily routines, it is seamlessly integrated with cognitive control that allows conscious override of the automatically planned motor sequences resulting in flexible behavior [8, 21, 22]. A variety of methods have been used to examine the physiological underpinnings of cognitive control and the associated temporal stages [21–28]. Nonetheless, the spatio-temporal stages of the interplay of automatic and controlled processing are still poorly understood. In an effort to contribute to this line of research, the current study uses a temporally sensitive method to track neural activity in real time during processing of incongruent trials that induce conflict on perceptual vs. motor levels. This approach makes it possible to examine the interplay between automatically generated motor plans and ad hoc executed response reversals indexing cognitive control.

The structure of the flanker task presents an opportunity to study the neural basis of different dimensions of incongruity processing with an emphasis on cognitive control over simultaneously activated, competing motor plans. Different task forms have been used extensively in fMRI and ERP studies [29–32]. The color task version used in the current study [17, 33, 34] presents irrelevant flanker squares in one of four colors on both sides of a central target square. The target is shown after a short delay and participants are instructed to ignore the flankers and respond to the color of the target. Because two different colors are mapped on each hand, the conflict between flankers and targets is manipulated in two ways. At the stimulus incongruity (SI) level, the two colors are perceptually different but they both map to the same hand and do not necessitate a change of response. At the response incongruity (RI) level, the flankers prime an incorrect response which must be switched to the correctly-responding hand following the target appearance. Both conflict conditions can be compared to congruous (CO) trials on which flankers and the target are the same color. The incongruity effect is further facilitated in the current study by displaying the flankers alone for a short period of time just before the target [35]. Previous evidence shows that accuracy is lowest and response times longest to RI trials confirming that response interference is greater than the perceptual interference [17, 32, 36]. Imaging evidence suggests that the medial prefrontal cortex, especially in the anterior cingulate cortex (ACC) but also pre-supplementary motor area (preSMA) are activated during response interference, in addition to lateral ventral and dorsal frontal areas [17, 29, 30]. This pattern is consistent with other types of tasks eliciting response conflict such as the Stroop [37–40] and error processing [41, 42] as this network is engaged during cognitive control tasks probing conflict detection and response selection.

Functional imaging provides spatial mapping of the involved areas but techniques with higher temporal precision that can resolve different stages of processing are needed to examine on-line activity changes during cognitive control engagement. Studies using EEG and MEG methods have investigated the oscillatory dynamics of movement preparation and the cognitive control influences as they unfold during conflict-evoking conditions. Imagined or actual engagement of the motor circuitry is reflected in oscillatory activity within beta frequency band (15–25 Hz). Beta power decrease, also termed desynchronization, is observed during movement initiation and execution and during imagined movements [43–45]. Beta decrease is generated bilaterally in the sensorimotor cortices with dominance contralateral to movement [46, 47]. This asymmetry has been exploited successfully in brain-computer interface systems [48]. Lateralized beta decrease measured over the sensorimotor areas in the two hemispheres is anticipatory in nature. It commences well in advance of a motor action [47] and is predictive of the upcoming movement. Cheyne and colleagues [25, 49] used a go/switch task which established response prevalence on the majority of trials and required a response switch on a subset of trials. Using MEG source modeling, they reported sensitivity of beta decrease to the laterality of response preparation to correct and erroneous switches. These features make beta oscillations well suited for investigating involuntary motor preparation induced by the presentation of flankers and the controlled override during deliberate motor decisions. They provide information on the relative timing and dominance of the engagement of the sensorimotor cortices in the two hemispheres.

Theta oscillations are sensitive to cognitive effort, which is reflected in increased event-related theta power in response to higher demands during the engagement of cognitive control [50–52]. Source modeling of MEG and EEG signal indicates that the ACC is a strong generator of theta during response conflict [16, 50, 53] with additional sources reported in the lateral prefrontal cortex [16, 51]. These estimates are consistent with intracranial recordings during cognitive tasks [54–56]. MEG studies investigating response stopping and switching have reported theta activity in the right anterior frontal cortex during both conflict conditions, suggesting that it subserves cognitive control more broadly [25, 49]. In a MEG study, McDermott and colleagues [57] used an arrow-based flanker task and reported an increase in theta synchronization in brain regions subserving cognitive control. Even though theta reflects different aspects of conflict in the response domain including response inhibition, selection, and action monitoring [50], it is not clear whether this extends to conflict in the stimulus processing as well. This was addressed by Nigbur and colleagues [32] who used a letter version of the flanker task in an EEG study. They reported event-related theta increase to both perceptual and response-related incompatibility. In addition, they calculated theta phase synchrony between electrodes over the medial and lateral fronto-central scalp regions and observed increased theta coupling during trials inducing response conflict.

The question of automatic vs. controlled processing is particularly germane to addiction. Animal research and human studies indicate that the circuits underlying cognitive control are especially vulnerable to addiction including alcoholism [58, 59]. Long term excessive alcohol use is associated with compromised structural and functional circuitry primarily in the frontal lobes resulting in dysregulation of cognitive functions [60–62]. In a series of previous studies, we have reported that alcohol intoxication selectively attenuates cognitive control, which is engaged during high levels of response conflict. In a BOLD fMRI study, we found that the ACC activity to high conflict trials on a Stroop task is selectively reduced under acute alcohol intoxication [39]. In a MEG study with the same task, Kovacevic et al. [16] found that alcohol reduced conflict-related theta activity in the ACC during early conflict detection, and late response selection stages. Moreover, similar effects have been observed across different tasks and motor effectors, and corroborated with complementary imaging methods [16, 17, 39, 41, 51]. This deficit may underlie inability to maintain inhibitory control over harmful levels of drinking which is considered to be a factor in subsequent alcohol abuse [59, 63–66]. However, it is not clear whether alcohol effects are specific to response conflict or whether they can be explained with attentional deficits in the stimulus-processing stream. Indeed, even relatively low alcohol doses lead to attentional impairments [67] which are reflected in deficient novelty detection [68]. Bartholow and colleagues [69] recorded ERPs during a flanker task in participants that were given placebo, low (0.4 g/kg) or high (0.8 g/kg) alcohol dose. They concluded that alcohol primarily impaired response inhibition but that the lower dose affected allocation of attention. However, the ERPs did not provide insight into spatial aspects of activity. Our fMRI study that used the color flanker task confirmed that alcohol exerts its influence mainly on response inhibition and selection [39].

The aim of the present study was to employ a multimodal imaging approach to further examine the spatio-temporal stages of processing conflict in the perceptual vs. response domains with the same flanker task as a function of alcohol challenge in a counterbalanced design. Our anatomically-constrained MEG (aMEG) method combines whole-head MEG and structural MRI within a distributed source model to estimate cortical sources in real time. Oscillatory dynamics of the MEG signal were analyzed as event-related power in beta and theta frequency bands. Changes in beta power were examined to reveal the hemispheric interplay of flanker-induced automatic processing and target-induced response reversal. Theta activity was analyzed to explore how the increased need for cognitive control is implemented across the cortex. Furthermore, we calculated co-oscillations between the principal nodes to examine conflict processing in real time at the level of an interactive system. We did so by analyzing phase locking values (PLV), a measure of the consistency of theta phase synchrony across time between the involved cortical areas [70].

## Methods

### Subjects

Eighteen young, healthy volunteers (11 men, mean [± SD] age = 25.1 ± 3.5 years, range 21–34 years) participated in this study and completed all sessions of the experiment. All participants were of legal drinking age in the United States. Participants were right handed, non-smokers and reported no medical, alcohol- or drug-related problems; no family history of alcohol or drug abuse in their first- or second-degree relatives; no medications at the time of the study; no previous head-injuries; and no MRI contraindications. Subjects reported drinking alcohol occasionally (2.0 ± 1.1 times a week) and in low-to-moderate amounts (2.7 ± 1.1 drinks per occasion) in social settings and did not have alcoholism-related symptoms. Written informed consent was obtained from all subjects before participation and subjects were reimbursed for their participation. Two additional women participated in the study, but their data were discarded due to a substantial number of noisy channels in one of the two experimental sessions. This study was approved by Partners Human Research Committee—Massachusetts General Hospital (protocol number: P-000181;MGH).

### Task

**Fig 1:**
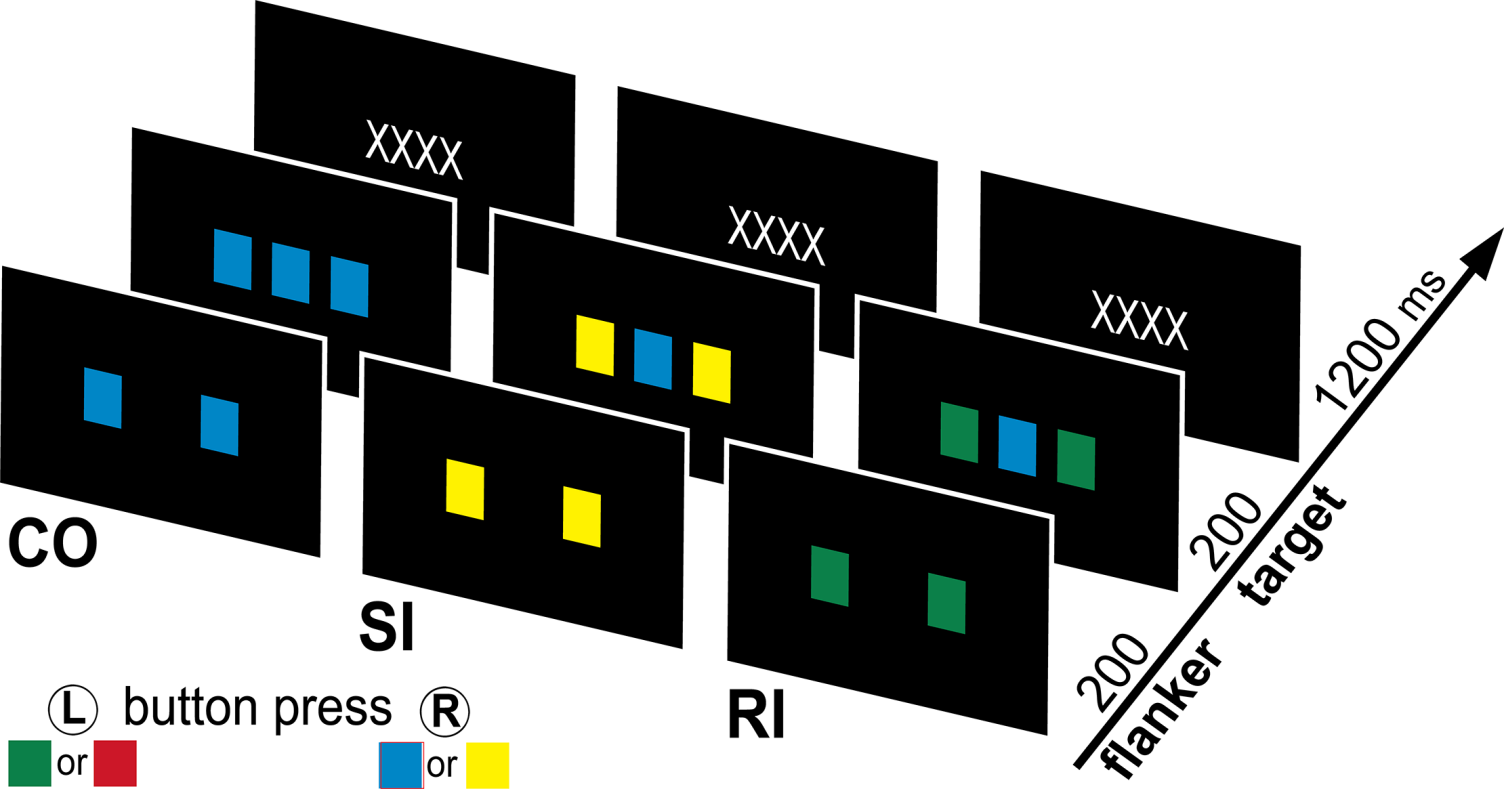
Schematic of the modified color version of Eriksen flanker task. Two flanker squares of the same color are presented for 200ms, followed by a central target square for 200ms. During the 1200ms fixation period, subjects are asked to respond to the color of the target using one of two buttons with their left (L) or right (R) index fingers, each corresponding to two of four colors. Congruent (CO) trials use the same color for flankers and target. Stimulus-incongruent (SI) trials have different colors, but both colors are mapped to the same button. Response-incongruent (RI) trials have different colors mapped to different buttons.

A color version of Eriksen flanker task [17, 33] was used in this study. Two flanker squares of the same color (red, green, blue, or yellow) were presented to the left and right of the central location for 200 ms (Fig 1). A target square was presented between the flankers for 200 ms, followed by a fixation string (“XXXX”) presented for 1200 ms. Participants were instructed to respond to the color of the target square by pressing the left button for a green or red target square and the right button for a blue or yellow target square, using their index fingers. On congruent (CO, 256 trials, 50%), the targets and flankers were the same color. The task introduced two levels of conflict when the target and flanker were displayed in different colors. On stimulus-incongruent (SI, 128 trials, 25%), the flankers and targets differed only in color but were both mapped to the same button. Alternatively, on response-incongruent (RI, 128 trials, 25%), the flanker and target colors were mapped to different buttons, requiring subjects to inhibit inappropriate responses elicited by the flankers. The RI and SI conditions comprised the same number of trials, and the color distribution was counterbalanced across trials. Overall, half of the trials were incongruous but the response switch was required only on RI trials. This pattern established a prepotent response which is commonly used in tasks probing motor control such as a go-nogo task [49]. A total of 512 trials were presented every 1600 ms using Presentation software (NeuroBehavioral Systems). Participants were instructed to respond as quickly as possible without sacrificing accuracy.

### Experimental design

All subjects first participated in an introductory session during which they were familiarized with the laboratory setting and the experimental procedure. Subjects subsequently participated in both alcohol and placebo MEG sessions in a counterbalanced manner. The within-subject design minimizes influence of individual differences in anatomy, alcohol metabolism, and brain activation patterns, resulting in reduced error variance and increased statistical power. Placebo and alcohol sessions were 33 ± 28 days apart on average. Urine pregnancy tests administered to women in the beginning of each session ascertained that none was pregnant. All subjects were asked to abstain from food for 3 hours and from alcohol at least 48 hours prior to each experimental session. Breath alcohol concentration (BrAC) was measured with a breathalyzer (Draeger, Inc.) throughout the recording session. The subjects rated their moods and feelings with the Biphasic Alcohol Effects Scale (BAES) [71] prior to drinking (at baseline) and on the ascending and descending BrAC limbs.

In each experimental session, either alcohol (0.60 g/kg for men, 0.55 g/kg for women, in a cocktail containing vodka (Grey Goose, Bacardi) as 20% v/v in orange juice), or placebo (the same volume of orange juice) were administered to the participants [41]. The task was administered close to the peak on the descending limb of the BrAC, as the average BrAC levels were 0.058 ± 0.012 before and 0.051 ± 0.010 after the task. Upon completion of each session the participants rated task difficulty, estimated the quantity of alcohol they drank, and reported how intoxicated or nauseous they felt. Transportation home was provided to all participants. High-resolution structural MRI scans were obtained from all participants in a separate session.

### Data acquisition and analyses

#### MRI

Structural MRI images were acquired on a 3T Siemens Trio whole-body scanner (Siemens, Erlangen). Two high-resolution 3D MP-RAGE T1-weighted sequences were obtained for each subject (TR = 2.53 sec, TE = 3.25 ms, flip angle = 7 degrees, FOV = 256, 128 sagittal slices, 1.33 mm thickness, in-plane resolution 1 x 1 mm) and used to reconstruct each person's cortical surface [72, 73]. The inner skull surface derived from the segmented MRI data was used for a boundary element model of the volume conductor in the forward calculations. The solution space was approximated by ~5000 free-rotating dipoles along the gray-white matter surface in the cortex, with spacing between dipole locations ~7 mm.

#### MEG

High-density MEG signals were recorded with a whole-head Neuromag Vectorview system (Elekta) in a magnetically and electrically shielded room. The signals were recorded continuously with 601 Hz sampling rate and minimal filtering (0.1 to 200 Hz). The location of head position indicator (HPI) coils, the main fiduciary points (i.e., the nasion and preauricular points) and a large array of random points covering the scalp were digitized with 3Space Isotrak II (Polhemus) system for subsequent precise co-registration with structural MRI images. MEG data analysis stream has been described in previous publications [16, 51, 53]. It primarily uses custom MATLAB functions and relies partly on publicly available packages including FieldTrip [74], EEGLab [75], and MNE [76].

Data from 204 channels (102 pairs of planar gradiometers) were analyzed in beta (15–25 Hz) and theta (4–7 Hz) frequency bands. Data from the additional 102 magnetometers were not included in the analysis. After wide-band filtering (0.1 to 100 Hz), data were epoched from -400 to 600 ms relative to each target onset for the stimulus-locked analysis and 300 ms before and after response (i.e., button press) for the response-locked analysis. For the beta band, analysis was carried out separately for each responding hand to examine laterality of the spatio-temporal stages of motor preparation and execution in greater detail. Independent component analysis was used to remove heartbeat and eyeblink artifacts [75]. Any remaining artifacts were removed with threshold rejection and careful visual inspection [74]. Baseline correction was performed on each epoch by averaging over a 200ms baseline preceding the flanker presentation and subtracting this average from each sample in the epoch. Only artifact-free trials with correct responses were included in the analysis. To eliminate potential bias due to unequal number of trials, trials were equated across beverage and task conditions for each subject by excluding randomly selected superfluous trials with an average of 85 +/- 15 trials remaining for each condition.

Complex power spectra were calculated for each trial with Morlet wavelets in 2 Hz increments for beta and 1 Hz increments for theta [70]. Padding was added to each epoch and then discarded to remove edge artifacts from wavelet analysis. Power plots for all trials were inspected for possible remaining artifacts. Empty room data pooled across sessions were used to estimate the noise covariance for the calculation of the inverse operator and to prevent biasing the inverse solution against spontaneous brain oscillations. Empty room data were band-pass filtered between 3 and 50 Hz. The noise covariance matrix was scaled by the regularization parameter, incorporating the estimate of signal-to-noise ratio (SNR) equaling 5 [77]. Identity matrix was used for noise-sensitivity normalization of the source-space solution. Source power estimates were obtained with a noise-sensitivity normalized anatomically constrained minimum norm model [78, 79] for both the beta and theta bands.

The estimates of source power and phase were obtained at each location on the cortical surface for each trial and at each frequency, and then averaged across band frequencies (beta: 15–25 Hz; theta: 4–7 Hz) and across trials for each condition. Finally, event-related power estimates were expressed as percent signal change from baseline calculated for the 200 ms period preceding the flanker. Group averages were created by morphing each subject’s reconstructed surface into an average representation by aligning their cortical sulcal-gyral patterns [80] and averaging individual source power estimates.

Region of interest (ROI) analysis was conducted to examine possible interactions of the factors of task condition and beverage on changes in beta and theta power. ROIs were created based on overall source power averages across all subjects, task and beverage conditions. They were drawn in an unbiased manner to comprise groups of dipoles along the cortical surface with most notable source power. The same set of group-based ROIs was used for all subjects in a manner blind to their individual activations via automatic morphing [80]. Timecourses for each ROI were extracted by averaging over all vertices included in the ROI and are presented as percent change from baseline. The ROIs primarily encompassed the cognitive control network in the medial and lateral frontal regions and the motor areas bilaterally, including the dorsal ACC, pre-supplementary motor area (preSMA) on the medial surface, the inferior frontal cortex (IFC), and sensorimotor regions (SMOT) in the peri-Rolandic area, in addition to the visual area in the medial occipital cortex (Occ). Prestimulus raw power did not differ across conditions or beverages for either beta or theta bands for any of the selected ROIs indicating that the observed event-related changes were not due to baseline differences.

Co-oscillatory interactions between cortical ROIs were estimated by calculating phase-locking values (PLV) [70, 81]. PLV is a measure of synchronization that is sensitive to the consistency of phase difference in a particular frequency range between two ROIs regardless of their amplitude [70].

Data were statistically analyzed across all ROIs with repeated measures ANOVAs with the factors of Beverage (alcohol vs placebo) and Condition as described in the relevant results sections below. A significance threshold (alpha) of 0.05 was adopted in all analyses. Bayes factors were also applied to determine the fit of the data under the null hypothesis versus the alternative hypothesis and are listed as BF values [82–84]. It has been suggested that a BF above 3 indicates substantial support for the alternative hypothesis. Conversely, a BF below 0.33 reflects support for the null hypothesis [83].

## Results

### Behavioral measures

**Fig 2:**
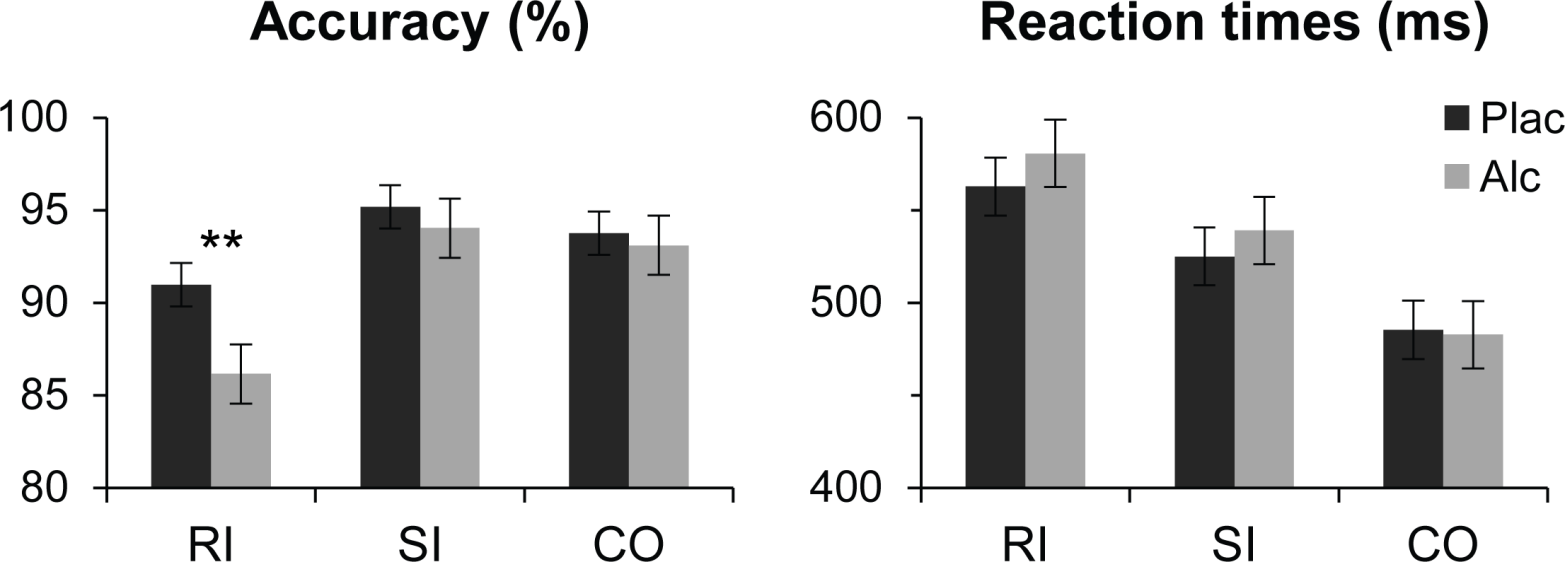
Behavioral results on flanker task. Response interference effect is reflected in lower accuracy and longer RTs on RI trials. Accuracy was equivalent on SI and CO trials, but stimulus-related incongruity resulted in longer RTs on SI trials. Alcohol intoxication decreased response accuracy selectively on RI trials.

As shown in Fig 2, a strong flanker-induced response interference effect on accuracy was reflected in a main effect of condition, F(2,34) = 14.41, p < 0.005, with lower accuracy on RI trials compared to all other conditions, F(1,17) = 14.99, p < 0.001, BF = 279.4, but no difference in accuracy between CO and SI trials. There was a strong main effect of Condition on RTs, F(2,34) = 106.91, p < 0.0001, all pairwise BFs > 3x10^10^, with slowest responses given to RI stimuli (572 ± 65 ms) followed by SI (532 ± 74 ms), and fastest responses to CO (484.82 ± 73 ms). Alcohol intoxication selectively affected response conflict as evidenced by decreased accuracy only on RI trials, F(1,17) = 8.43, p < 0.01, BF = 12.6, and by longer RTs to RI relative to CO/SI under alcohol compared to placebo, F(1,17) = 6.26, p < 0.05, BF = 4.5. Accuracy correlated negatively with impulsivity scores, r = -0.62, p < 0.01.

Participants reported feeling more stimulated, high, and euphoric under alcohol than placebo, with a significant beverage effect on the aggregate of these variables t(1,17) = 3.427, p < 0.005, BF = 33.2 on the ascending BrAC limb. The average of these self-reports of stimulation correlated with BrAC, r = 0.7, p < 0.01. On the scale from 1 (not at all) to 5 (very much), participants reported feeling moderately intoxicated (2.7 ± 0.7) under alcohol, but not at all intoxicated under placebo (1.0 ± 0), t(1,17) = 10.83, p < 0.001.

### MEG results

#### Beta band

**Fig 3:**
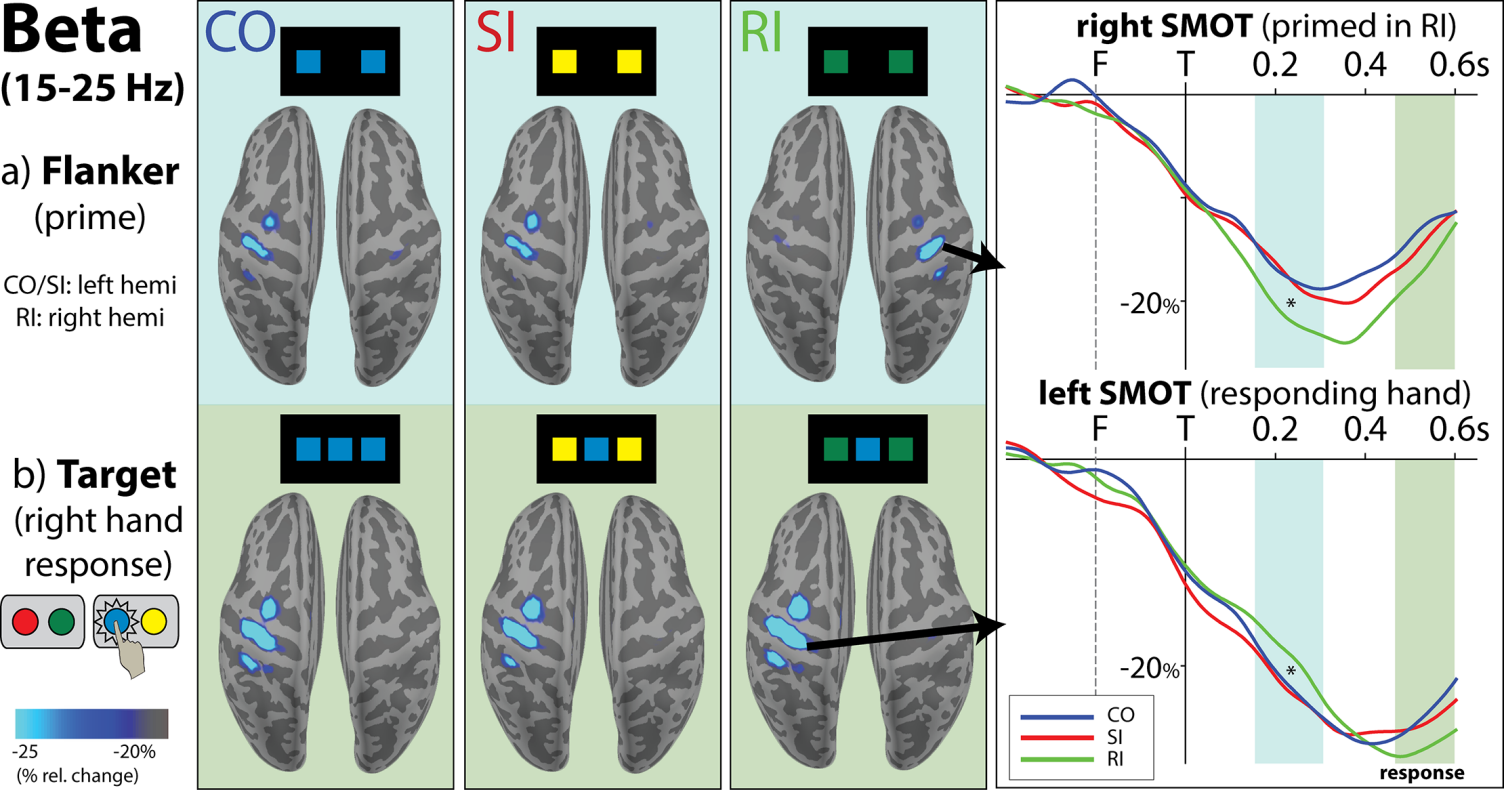
Activation maps and timecourses for event-related beta power expressed as percent change from pre-flanker baseline. ‘F’ and ‘T’ correspond to flanker (at -200 ms) and target (0 ms) presentation respectively. Beta power in the early time window (150-300ms), highlighted in blue, is sensitive to response preparation primed by the flanker: in this example, CO and SI prime the left hemisphere (correct response hand), as shown with greater beta decrease (timecourse shown in bottom right panel). In RI, the right hemisphere is incorrectly primed by the flanker as shown with beta decrease to RI (timecourse shown in upper right panel). Upon the presentation of the response-incongruent target, the response preparation is switched to the opposite hand to make a correct response. During the late time window (450-600ms), highlighted in green, stronger beta decrease in the correctly-responding left hemisphere is seen in all conditions. Therefore, the relative changes in beta power track motor preparation in real time. Only correct trials requiring a right-hand response are shown here, but the findings on left-hand trials are a mirror image of the pattern shown above.

It is well known that beta power decreases during preparation and execution of movement in bilateral sensorimotor cortices with greater attenuation in the contralateral areas [47, 85]. As shown in Fig 3, the downward deflection of beta power begins in anticipation of the impending movement in sensorimotor areas of both hemispheres and decreases to about -40% of the total beta power change by the time of target presentation. On RI trials, there is a mismatch between the response hand primed by the flanker and the hand indicated by the target. The subsequent relative changes in beta power track switching in motor preparation and provide insight into decision making in real time. The switching process that occurs on RI trials is illustrated over the time window of 0ms (presentation of target) to 600ms in the supplementary video (S1 Video) online.

#### Automatic, flanker-induced response preparation

A priming effect induced by the flanker is seen during the early time window, here tested within 150–300 ms (Fig 3). Statistical comparisons were carried out on data collapsed across both responding hands, with hemispheres defined as ipsilateral or contralateral to the responding hand. The effects of response incongruity necessitating switching were assessed by comparing beta activity averaged over CO/SI to beta on RI trials. The results for each responding hand were independently significant and were a mirror image of each other. For CO and SI trials, the hemisphere that is primed by the flanker is the same hemisphere that executes the response, i.e. the primed hemisphere is contralateral to the responding hand. Therefore, there is more beta decrease to CO/SI trials in the contralateral hemisphere at this time than to RI trials, F(1,15) = 21.09, p < 0.001, BF = 4900.6. Conversely, on RI trials the ipsilateral hemisphere is incorrectly primed by the flanker, resulting in more beta decrease to RI compared to CO/SI, F(1,15) = 11.26, p < 0.005, BF = 49.0. The observed relative differences in beta power to RI confirm that motor preparation is triggered automatically by the flankers despite instructions to disregard the flankers and to respond only to targets.

#### Target-induced countermand on RI trials

However, the target presentation on RI trials countermands the motor plan as the activity is switched to the opposite hemisphere/response hand. The time window of 450-600ms, highlighted in green in Fig 3, encapsulates the average response time for all conditions. During this time window, greater beta power decrease in the responding SMOT cortex during RI trials reflects correct response execution.

#### Response-locked beta estimates

**Fig 4:**
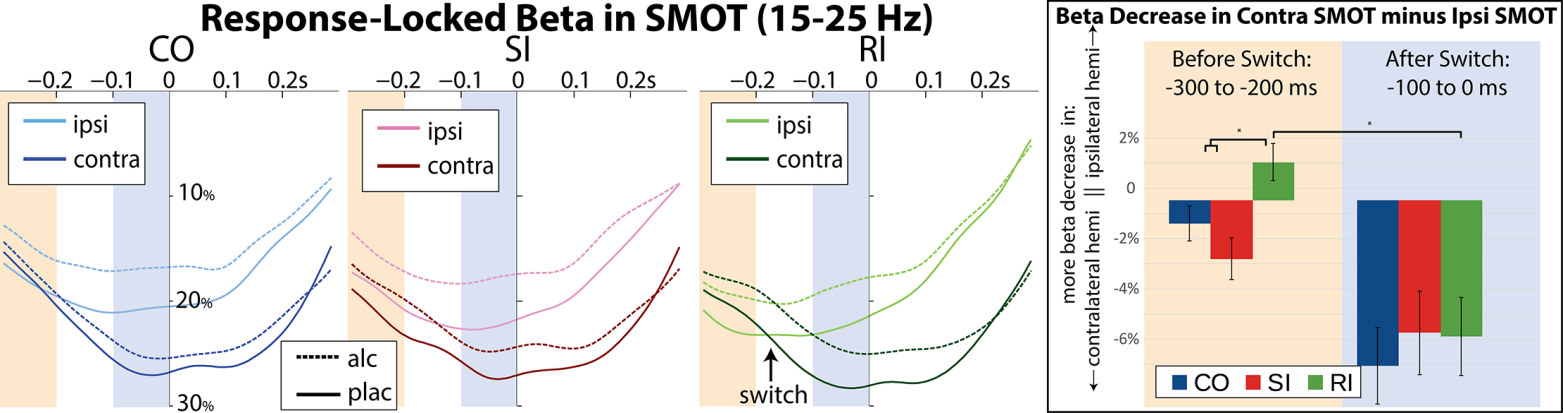
Beta power timecourses time-locked to the response (button press at time zero), expressed as percent change from baseline in the SMOT areas. Darker colors in each condition signify the contralateral hemisphere to the responding hand, which always has more beta decrease than the ipsilateral hand (lighter color) at the time of the response. On RI trials, the early time window of -300 to -200ms pre-response (highlighted in peach color) shows that the ipsilateral (primed) hemisphere has more beta decrease, but the two hemispheres switch at 173 ms before the response, marked by the arrow. After the switch and immediately preceding the response (highlighted in dark blue color), the primed hemisphere rebounds back to baseline while the responding hemisphere continues to decrease. In the right panel, lateralized beta power is presented in the bar graphs as a subtraction of beta power in the contralateral—ipsilateral SMOT in the early (-300 to -200 ms) vs late (-100 to 0 ms) time windows.

To further examine timing of the switch of the hemisphere engagement on RI trials, beta source power was calculated with respect to button-press responses and expressed as percent change from the pre-flanker baseline. Timecourses for the two SMOT areas were overlaid to see relative timing of beta decrease in each hemisphere (Fig 4). For RI trials, flankers incorrectly prime ipsilateral SMOT cortex resulting in a greater early beta power decrease. However, a switch occurs at -173 ms before the response on average, as indicated by greater beta decrease in the responding (contralateral) hemisphere as it prepares to execute the actual response. Therefore, beta power makes it possible to track the relative engagement of the two hemispheres as a response decision is made and the automatic preparation is reversed. The switch between the two hemispheres was observed only on RI trials under both placebo and alcohol. To compare this effect across conditions, ANOVA was performed on the subtractions of relative change in the contralateral minus ipsilateral hemispheres, as shown in the bar graph in Fig 4. There was a main effect of condition in the time window before the switch in the subtractions of the contra- minus ipsilateral beta power in SMOT (CO/SI vs. RI), F(1,15) = 9.01, p < 0.01, which was comparable and significant in each beverage separately (placebo: F(1,15) = 7.04, p<0.05, BF = 6.8; alcohol: F(1,15) = 6.91, p < 0.05, BF = 6.4). The only effect of alcohol was attenuated beta desynchronization compared to placebo overall, F(1,15) = 19.59, p < 0.0005, BF = 14.7. No interactions between beverage and conditions were observed, suggesting that beta is not susceptible to differential effects of incongruity as a function of intoxication.

### Theta band

**Fig 5:**
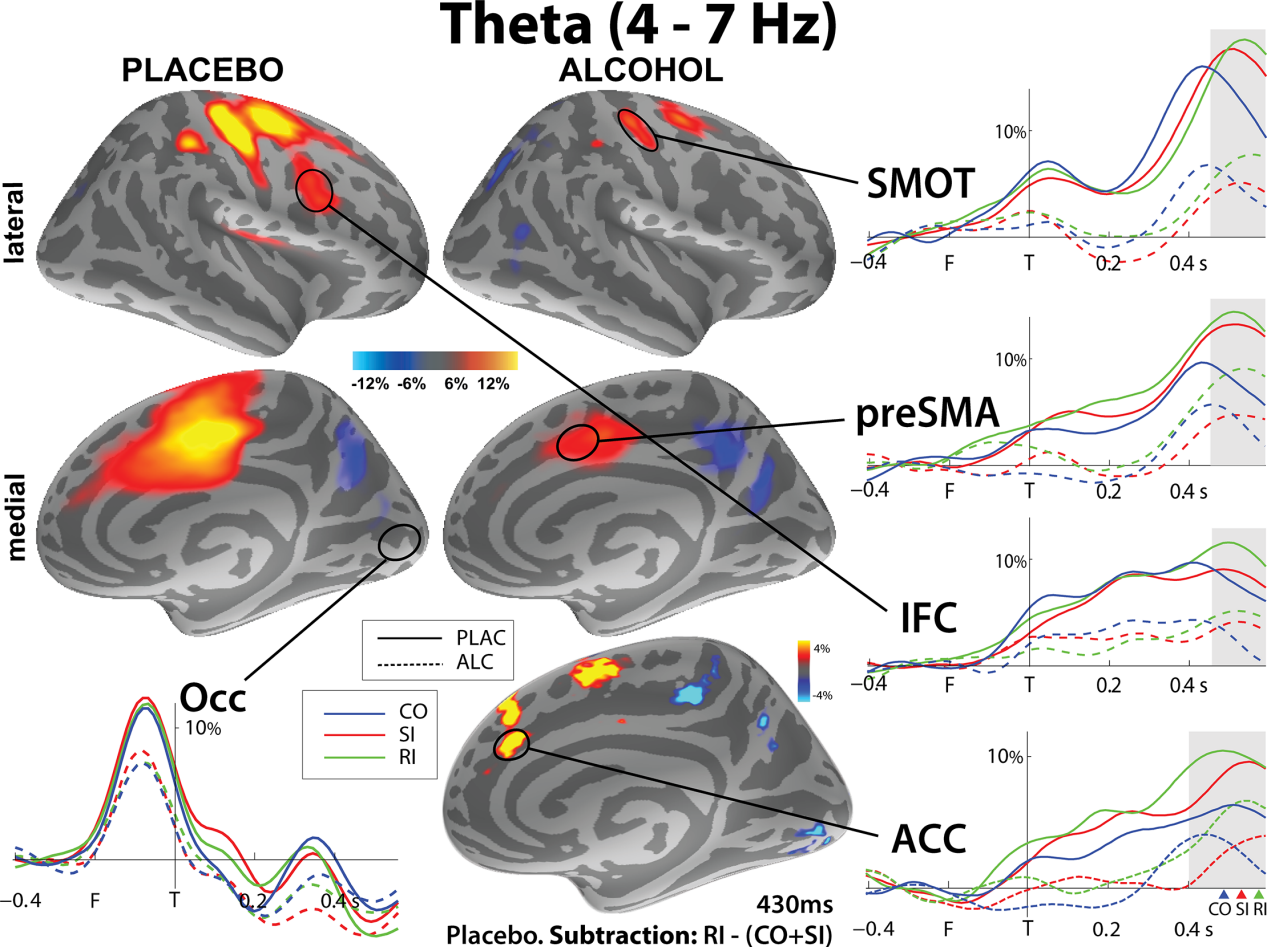
Group-average maps of event-related theta source power estimates and the associated timecourses, expressed as percent change from pre-flanker baseline. ‘F’ and ‘T’ correspond to flanker (at -200 ms) and target (0 ms) presentation respectively and colored arrows mark average reaction times for CO, SI and RI respectively. Gray windows highlight the peak activity across ROIs (450-600ms for most ROIs and 400-600ms for ACC to account for earlier activation to RI). Under placebo, sensorimotor cortex (SMOT) and pre-supplementary motor area (pre-SMA) show higher theta to both types of incongruity, while right inferior frontal cortex (IFC) and anterior cingulate cortex (ACC) are especially responsive to RI trials. Alcohol abolished sensitivity to both types of incongruity in SMOT, but retained higher theta to response incongruity in pre-SMA. Theta increase to RI trials was abolished in the IFC under alcohol, but preserved in the ACC.

Theta activity followed an expected spatio-temporal pattern (Fig 5) in the form of dynamic spatio-temporal maps and associated timecourses. The earliest event-related theta power increase was observed in the visual cortex at ~120ms after the presentation of flankers. While there were no differences between conditions at that time, F(1,17) = 1.25, n.s., a main effect of beverage, F(1,17) = 4.49, p < 0.05, indicated alcohol-induced decrease in theta power. A visual off-response is additionally visible in the occipital cortex at ~330ms after target presentation. Subsequent event-related theta power increases were estimated mainly to the medial and lateral frontal areas that show regionally specific sensitivity to condition types. The effects of condition and beverage were tested in the 450-600ms time interval after target presentation. Overall, theta power was greatly reduced under alcohol compared to placebo with a main effect of beverage in all ROIs during peak activity, all F’s > 8.9, p < 0.01.

The flanker task made it possible to examine effects of incongruity overall by contrasting an average of both types of incongruity to congruous trials, (SI+RI vs CO) and to also compare the two types of interference (SI vs RI). Under placebo, the cortical areas subserving motor control were sensitive to incongruity including bilateral SMOT, F(1,17) = 5.95, p < 0.05, and right preSMA, F(1,17) = 11.39, p<0.01. The right IFC was preferentially sensitive to response conflict, F(1,17) = 5.38, p < 0.05; BF = 3.0. In the right ACC, response conflict also evoked greater theta across beverages as reflected in a main effect of condition: F(1,17) = 6.91, p < 0.05, BF = 6.2. However, the RI theta peaked earlier under placebo (~440 ms) than under alcohol (~475 ms), t(1,17) = 2.45, p < 0.05.

Alcohol abolished sensitivity to both types of incongruity in bilateral SMOT, F(1,17) = 1.51, n.s.; BF = 1.5, while the right preSMA maintained sensitivity to response incongruity only F(1,17) = 9.66, p < 0.01; BF = 22.6. The rIFC no longer differentiated between the two types of conflict under alcohol, F(1,17) = 0.58, n.s., BF = 0.31, but overall incongruity (SI+RI) evoked greater theta compared to CO, F(1,17) = 10.99, p < 0.01, BF = 24.4.

Theta in the SMA, ACC and IFC correlated negatively with reaction times on conflict trial, i.e., subjects with greater theta had faster RTs in SI+RI under placebo (ACC r = -0.7; IFC r = -0.55; SMA r = -0.49, all p < 0.05), but not under alcohol (all r’s > -0.2). Under alcohol, theta on RI trials in the ACC and preSMA correlated positively with impulsivity scores (all r’s > 0.49, p < 0.05) Similarly, theta in IFC on SI+RI trials correlated with disinhibition scores (all r’s > 0.49, p < 0.05).

#### Co-oscillations (PLV) in theta band

**Fig 6:**
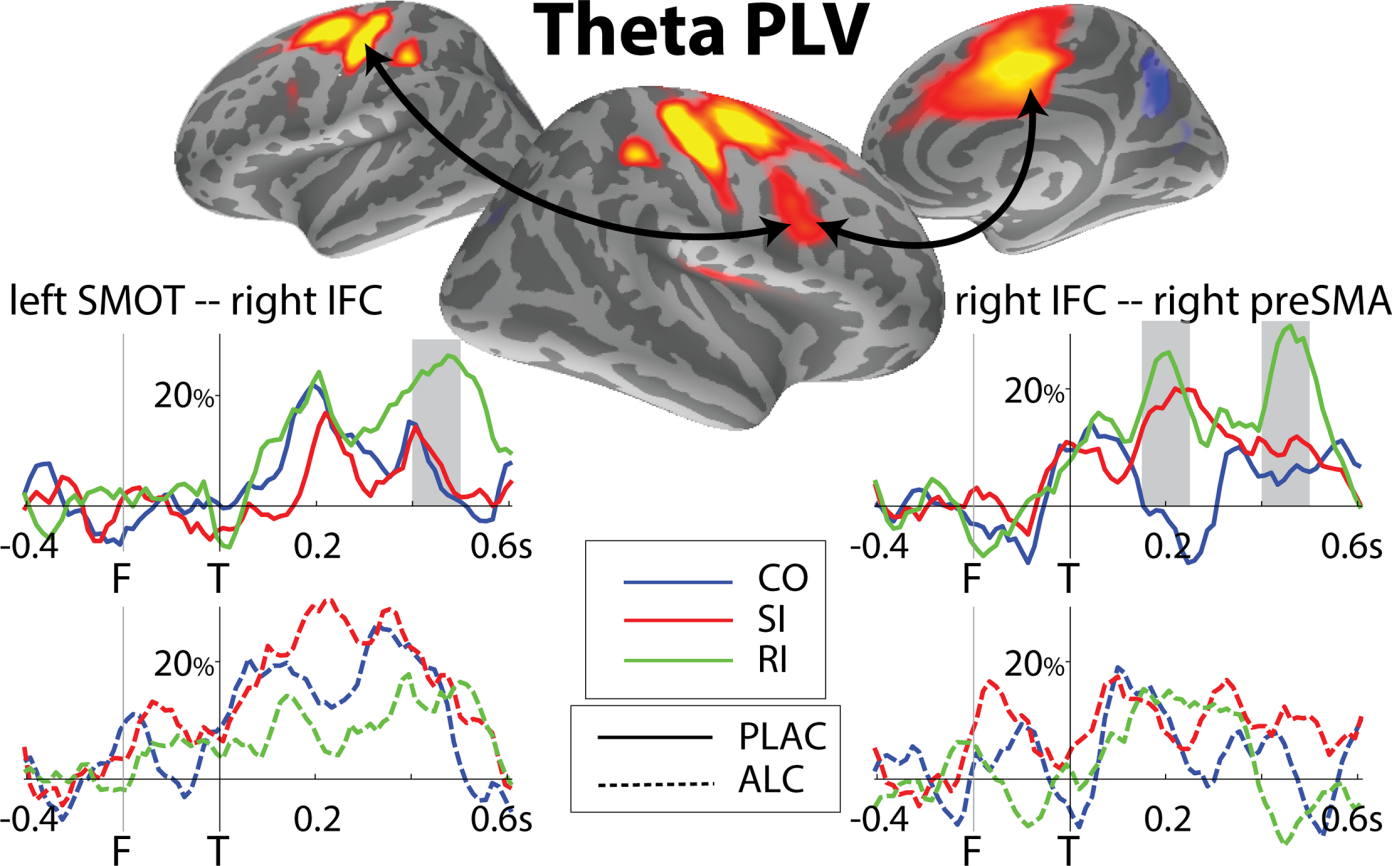
Group average maps and timecourses of phase-locking values across pairs of ROIs in theta band. ‘F’ and ‘T’ correspond to flanker (at -200 ms) and target (0 ms) presentation respectively. PLV is expressed in percent change from baseline. The right IFC shows increased synchronization with the lateral (SMOT) and medial (preSMA) motor regions. In the earlier time window (150–250 ms), higher PLV is found between the right IFC and right preSMA to both types of incongruity. In the later time window (400–500ms), the IFC co-oscillates with the SMOT and preSMA on RI trials only, indicating their engagement in response execution under conditions of response switching. Alcohol dysregulates co-oscillations between both pairs of ROIs.

To investigate effects of incongruity on functional interactions in real time and at the neural systems level, we estimated the degree of co-oscillations by calculating phase-locking values expressed in percent changes from baseline (Fig 6). Under placebo, the degree of theta phase-locking in the right IFC and SMOT areas increased transiently to all three task conditions at ~200ms. This was followed by a significant increase only on RI trials during response preparation (400–500 ms), F(1,17) = 6.94, p < 0.05, BF = 6.4, but the effect was abolished under alcohol, F(1,17) = 0.05, n.s., BF = 0.25. Between the right IFC and right preSMA, PLV in both SI and RI increased relative to CO at the earlier time window of 150-250ms, F(1,17) = 11.31, p < 0.005, BF = 49.7. Additionally, PLV increased to RI trials during the later time window, F(1,17) = 6.66, p < 0.05, BF = 5.6, indicating engagement of these areas in response planning under placebo. No significant co-oscillations between right IFC and right preSMA were observed under alcohol (early: F(1,17) = 0.28, n.s., BF = 0.25; late: F(1,17) = 3.69, n.s., BF = 1.38).

## Discussion

In the present study, we used an anatomically-constrained MEG approach to examine spatio-temporal stages of the interplay between automatic and controlled processing at two different levels of incongruity in a flanker task. Lateralized beta power decrease to flankers confirms that they activate motor plans automatically. Additionally, timecourses of beta oscillations provide timing insight into response switching on RI trials as cognitive control is engaged to override automatically activated motor plans. Theta oscillations are sensitive to the level of conflict as the medial (pre-SMA) and ventrolateral prefrontal (IFC) cortices were especially activated by the response incongruity, consistent with other evidence of their essential role in cognitive control. Synchronous co-oscillations between the medial and lateral prefrontal areas measured with PLV further reveal their integrated task-relevant contributions to inhibition and response switching. Acute alcohol intoxication had a major attenuating effect on theta oscillations overall and exerted differential effects on response conflict across prefrontal regions suggesting partial re-sculpting of their relevant engagement by task demands. Alcohol attenuated beta desynchronization overall in agreement with its known effects on inhibitory signaling, but beta was not susceptible to differential effects of alcohol on incongruity processing.

The color flanker task successfully manipulated conflict on both perceptual and response incongruity levels. RTs increased in a step-wise manner, with longer responses on SI compared to CO condition and the longest RTs to the response conflict on RI trials. However, the accuracy was equivalent on the CO and SI trials. Because the correct responding hand is primed by the flankers on both CO and SI trials, there is no change in response mapping which results in near-perfect accuracy on both conditions. Longer RTs on SI than CO trials can be interpreted as the cost of processing the stimulus-level incongruity in accordance with response rules. Impaired speed and accuracy on RI trials confirm that motor plans were activated automatically by the flanker stimuli without direct conscious control [5, 6] and indicate the additional processing time needed to implement countermanded motor plans and maintain adequate accuracy. Response conflict induced stronger interference than stimulus incongruity, in agreement with previous reports [17, 32, 36]. Alcohol intoxication selectively exacerbated response interference consistent with its effects on decision making circuitry under response conflict conditions imposing higher cognitive demands [16, 39, 51].

In the current study, high temporal resolution of the aMEG method provided insight into automatic priming and subsequent engagement of controlled processing in the context of the stimulus- vs. response-level conflict manipulation by the flanker task. Beta oscillations effectively tracked the unfolding of this process in real time and with good precision over both hemispheres. It has been well established that movement planning and execution are accompanied by a decrease of event-related beta power [43]. This decrease is observed bilaterally over the sensorimotor cortices but it is dominant over the hemisphere contralateral to movement [46, 47]. Our results confirmed an early, anticipatory decrease of beta power (Figs 3 and 4) indicating flanker-induced automatic motor priming. On CO and SI trials, the hand primed by the flanker is the same as the hand used to respond and no switch is required. Therefore, the flanker-induced motor preparation proceeds automatically until appearance of a target engages cognitive control. On RI trials, the automatic preparation is reflected in greater beta decrease in the “wrong” hemisphere. The target presentation then initiates a switch from the incorrectly primed to the “correct” hemisphere. This switch can be seen as a strong beta decrease in the responding hemisphere which dips lower than on CO and SI trials. This finding is consistent with beta sensitivity to the level of response conflict [86].

We explored the timing of this switch by computing response-locked beta oscillations over sensorimotor areas in both hemispheres. At approximately 173 ms before the response, beta power in the responding hemisphere reaches greater beta decrease than the primed hemisphere as it rebounds up towards baseline (Fig 4). This change in lateralization indicates a reversal in response preparation and selection of new motor plans to make the correct response. This timing aligns with the time needed to issue the countermanding command and execute the response based on electromyographic (EMG) data [87]. A direct comparison of beta power between the two hemispheres referred to as “lateralized beta power” (Fig 4) was inspired by studies of lateralized readiness potential (LRP), an index of hemispheric asymmetry over the sensorimotor cortex during motor preparation [88, 89]. EEG studies measuring LRP in flanker tasks find that RI trials initially activate an incorrect response which is then appropriately switched on correct trials [28, 35, 90]. Automatic initiation of motor plans has been confirmed with lateralized response fields in MEG studies using a go/switch paradigm by Cheyne and colleagues [49] who showed that beta suppression over the contralateral sensorimotor cortex reflects anticipatory activity and is predictive of correct motor preparation. Studies using EMG recordings of hand muscles confirm subthreshold activation of motor responses that are suppressed prior to the actual execution [87]. Indeed, in the present study, direct comparison of the relative beta power recorded over the left and the right sensorimotor regions revealed that the incorrect motor preparation decreases simultaneously with increasing of the correct motor activation. This is consistent with a gradual tradeoff in motor excitability between the two hemispheres as shown in studies combining stimulation and electrical recordings of brain and muscle activity [27, 28]. Beta oscillations are the preferred frequency characterizing activity in the motor cortex and result from functional interactions between the cortex and the basal ganglia, with the cortex having a leading role in those interactions [91].

Our findings are strongly supportive of the automaticity and interdependence of stimulus and motor processing. Although the participants were instructed to “disregard the flankers”, they were unable to do so deliberately and avoid automatic motor activation [17, 32, 35]. The parallel processing of automatic versus instructed behavior extends to other domains and reflects the generality of the brain’s cognitive control networks in responding to diverse aspects of motor processing. It has been demonstrated in numerous studies probing a range of functions including oculomotor control [92], and object affordance [93]. Application of transcranial magnetic stimulation (TMS) along with ERPs and MEPs has provided complementary evidence on changes in motor excitability and the relative timing of engagement of cognitive control and switching of response plans [26, 28, 94–96]. Furthermore, studies of language indicate that words automatically trigger generic processing for meaning as it is impossible to decide not to understand a familiar word in one’s own language [97]. Therefore, most stimuli that we encounter activate relevant generic processing streams without conscious input. However, motor plans are monitored continuously in the context of changing environmental demands and can be modified in an integrated manner by regulative, anticipatory mechanisms [49, 98]. This proactive action framework has several advantages: advanced motor preparation assures primed and therefore faster responses especially under predominantly predictable circumstances; it comprises a range of motor actions or affordances that are situationally possible [99, 100]; motor plan modifications are smooth and integrated with a wider framework; furthermore, it is faster to initiate the most likely motor response in advance and make corrections as needed [101] than to start motor preparations after a stimulus has been processed. Therefore, along with other evidence, our findings support a broader ecological view of behavior which proposes that the motor preparation is triggered automatically and largely unconsciously,in a manner that is intertwined with input evaluation and is inherent to behavioral actions [4–6, 102]. This is consistent with the concept of parallel processing which has been interpreted with dual processing accounts [9, 10] and has been variably ascribed to separate or interdependent and overlapping neurofunctional pathways [8, 22, 26, 57, 103].

The current study employed acute alcohol challenge to examine its effects on the interplay of automatic and controlled processing. The importance of cognitive control on the ability to refrain from excessive drinking has been well established [58, 63–65], along with impairments of the frontal lobe associated with chronic alcoholism [60–62]. In a series of studies we have shown that the regulative top-down functions are particularly vulnerable to acute alcohol intoxication including the Stroop interference [16, 39], saccadic conflict [41], a modified Simon task [51], a color flanker task [17], and error processing [39, 41]. Behavioral results indicate that alcohol has particularly deleterious effects on response conflict, consistent with previous reports [16, 39, 69]. The aMEG model used in the present study is particularly suitable for investigating neuropsychopharmacological effects of alcohol [104], because MEG reflects the neural activity directly and is not susceptible to the confound of vasodilatory effects of alcohol which may affect the BOLD signal [105]. The present study indicates that the effects of alcohol on beta oscillations are rather subtle and generalized and are not dependent on response conflict. Alcohol did not affect the motor switching process which was analogous for both beverage conditions in its timing and amplitude. However, beta power decrease was less pronounced under alcohol overall which reflects its inhibitory effects via facilitation of the GABA_A_ receptor function [106, 107]. A similar effect of weaker beta desynchronization in motor areas was found in a MEG study in which a benzodiazepine was administered to healthy adults [108]. It is known that benzodiazepines are also GABA_A_ agonists and that they show cross-tolerance with alcohol, indicating that they affect common mechanisms [109, 110]. In contrast, theta oscillations showed pronounced vulnerability to alcohol intoxication in the present study in agreement with previous reports [16, 51, 53], suggesting that alcohol primarily affects cognitive control functions.

Increased theta is associated with cognitive control [16, 25, 50, 51]. It is seamlessly integrated with automatic processing as it tracks cognitive demands and is engaged proactively in situations involving ambiguity or indicating a need for response override [16]. In the present study, the primary generators of theta were estimated to the medial prefrontal cortex including preSMA and ACC, in the ventrolateral frontal cortex (IFC), and in the sensorimotor cortex (Fig 5). These spatial estimates are consistent with previous findings from other executive tasks such as the Stroop or a modified Simon task obtained with this method [16, 51] as well as with fMRI studies [37, 39] and intracranial EEG recordings [54–56]. Under placebo, theta power estimated to motor regions including the preSMA and SMOT was greater to both types of incongruity indicating that these regions are primarily concerned with motor intention and execution, but do not seem to be involved in the delineation of stimulus vs response incongruity [111]. In contrast, the IFC and the ACC responded preferentially to response incongruity under placebo. Both of these areas are known to play essential roles in a predominantly prefrontal cognitive control network during response conflict and the decision of how to respond seems to be addressed by these regions [112, 113]. IFC activation has been associated with planning internally driven movements which supports its essential contribution to maintenance of rule representation and action inhibition and selection [13, 112, 114]. A recent MEG study further showed importance of the IFC for coordinating long-range connections necessary for visuo-motor integration during a precision grip task [115]. Its activity is coupled with that in the ACC which may subserve efforts to execute actions in agreement with intents and goals [116] and which is sensitive to both early and late conflict processing stages and motor preparation [16]. The present data suggest that the IFC and ACC play a key role in the effective cancellation of automatically initiated but incorrect motor plans and executing adjustments to maintain performance accuracy. However, while alcohol challenge abolished the IFC sensitivity to response incongruity, theta increase to RI trials was preserved in the ACC under alcohol. This suggests that the ACC in particular subserves response incongruity which is consistent with its key role in movement planning and selection [117]. Similarly, under alcohol, the neighboring pre-SMA area maintained sensitivity to response incongruity only. The pre-SMA has been implicated in conflict-related activity and in initiating voluntary, intentional actions especially under situations demanding more complex control [103, 111, 118, 119]. The observed changes in regional sensitivity to response conflict under alcohol challenge suggests that the network may be slightly re-sculpted with increased contribution by the pre-SMA to action selection. This is consistent with previous studies implicating the pre-SMA in response selection, programming, and implementing task rules [22, 120]. It is known that alcohol intoxication increases cognitive demands during conflict tasks [51] and may necessitate additional resources to comply with task requirements. Therefore, the increased sensitivity of the pre-SMA to response conflict during alcohol challenge could reflect its compensatory activation to countervail alcohol-induced degradation of the cognitive control network [121]. A study of patients with traumatic brain injury reported that worse behavioral performance on a switching task was associated with greater activity in pre-SMA and IFC, as well as deficient connectivity between these areas [122].

Theta oscillations may represent the fundamental mechanism of integrating task-relevant information across different cortical domains [55, 123]. The cortico-limbic and cortico-cortical interactions are necessary for integrating plans and actions and for the top-down regulative input [124, 125]. In an effort to examine how the principal activated areas interact in real time and at the level of a system, we calculated phase locking values (PLV) on single trials. PLV is a measure of co-oscillations based on the phase similarity in a particular frequency range between two ROIs regardless of their amplitudes [70]. PLV increases transiently at times when it may be expected that different brain locations interact [81, 126]. Importance of the connection between the lateral and the medial prefrontal cortices for successful performance on tasks evoking cognitive control is well established [127–130] and is supported by their neuroanatomical connections [131]. The network-based view proposes that behavioral inhibition is an emergent property of an interactive network rather than being subserved by a single module [132]. As shown in Fig 6, the right prefrontal cortex (IFC) co-oscillates with the motor cortex and preSMA in a manner sensitive to both levels of conflict which aligns with its importance for long-range coordination [115]. The PLV was greater on RI trials at the time of response, suggesting that theta interactions underlie response reversal consistent with previous reports [32]. However, the network interactions are dysregulated by alcohol. This indicates that the fine tuning of the distributed neuronal envelopes that relies on the excitatory-inhibitory balance is disturbed by alcohol’s pharmacological effects. Alcohol-related wideband reductions in phase-locking have also been shown in both rats [133, 134] and humans [133] during an auditory oddball task.

In sum, by resolving the multiplexed MEG signal into beta and theta bands, we were able to examine the interplay between automatic and controlled processing during a flanker task. Hemispheric laterality of beta band changes in response to flankers and subsequent targets made it possible to track switching of response hands in real time. In contrast, theta oscillations estimated to medial and lateral prefrontal cortex were induced by both types of incongruity. The ACC and IFC were particularly sensitive to response incongruity, indicating their involvement in response inhibition and switching under placebo. Alcohol dysregulated this pattern and resulted in a shift towards greater activity of the pre-SMA during response conflict, while ACC remained sensitive to response incongruity across both beverages. Beta desynchronization was reduced under alcohol overall in agreement with alcohol-induced enhancement of inhibitory signaling. However, alcohol did not affect switching process. This type of research can have potential applications in further refinement of brain-machine interfacing for the purpose of using these indices for fluid decoding of automatic motor planning and moment-to-moment changes in cognitive control of intended motion.

## Supporting information

S1 Video Spatiotemporal dynamics of motor switching. Event—related beta power in bilateral sensorimotor cortices is displayed across time for each condition during right-hand response. A time range of -200 to 600ms is shown. Headings dictate when flankers and targets were on display and the time is displayed in the bottom left corner. In CO and SI conditions, the responding left hemisphere has more beta decrease throughout the epoch; conversely, in the RI condition, the incorrectly primed right hemisphere has more beta decrease initially, then switches to the left hemisphere when the correct response is made.
(MPEG)
